# Digital financial inclusion, industrial structure and urban–Rural income disparity: Evidence from Zhejiang Province, China

**DOI:** 10.1371/journal.pone.0303666

**Published:** 2024-06-27

**Authors:** Changcun Wen, Yiping Xiao, Bao Hu

**Affiliations:** 1 Institute of Rural Development, Zhejiang Academy of Agricultural Sciences, Hangzhou, Zhejiang, China; 2 School of Economics, Wuhan Textile University, Wuhan, Hubei, China; Gebze Teknik Universitesi, TURKEY

## Abstract

Rising income inequality challenges economic and social stability in developing countries. For China, the fastest-growing global digital economy, it could be an effective tool to promote inclusive development, narrowing urban–rural income disparity. It investigates the role of digital financial inclusion (DFI) in narrowing the urban–rural income gap. The study uses panel data from 52 counties in Zhejiang Province, China, from 2014 to 2020. The results show that the development of DFI significantly reduces rural–urban and rural income inequality. The development of DFI helps optimize industrial structure and upgrade the internal structure of agriculture, facilitating income growth for people in rural areas. Such effects are greater in poorer counties. Our findings provide insights into why rapid DFI and the narrowing of the rural–urban income disparity exist in China. Moreover, our results provide clear policy implications on how to reduce the disparity. The most compelling suggestion is that promoting the optimization of industrial structure through DFI is crucial for narrowing the urban–rural income gap.

## Introduction

Rising income inequality has become a core theme in social sciences, as it challenges economic and social stability [1]. For example, since 2020, the wealthiest 1% of the world population has captured almost two-thirds of all wealth [2]. More importantly, many developing countries have experienced severe income inequality within the last decade [3], which is primarily caused by the urban–rural gap [4]. Therefore, tackling the urban–rural income gap is significant for many developing countries. The growth of the digital economy has become important for economic growth in developing countries, especially in China. The worth of China’s digital economy soared from 11 trillion yuan ($1.6 trillion) in 2012 to 45.5 trillion yuan in 2021, accounting for 39.8 percent of the country’s GDP in 2021. At the core of the digital economy is digital finance which has become a rapid growth sector in China. It optimizes resources, promoting economic growth [5]. Can the development of digital finance alleviate the urban–rural income gap commonly faced by developing countries? Surprisingly, empirical evidence is rare.

Financial market failure plays a crucial role in the continued slow growth of rural income [6], especially in developing countries lacking financial infrastructures, causing low-income people to be denied access to formal financial services. Therefore, it is generally believed that financial repression and credit allocation polarization exists in developing countries [7–9], which intensifies the unequal distribution of national wealth and forms a vicious circle [10, 11]. According to World Bank estimates, around 60% of adults in developing economies, do not have access to any formal financial services [12]. To address these problems, the United Nations proposed the concept of inclusive finance for the first time in 2015, which aims at financial inclusion and sustainability and advocates that all members of society should have access to high-quality financial services at a reasonable price, especially the poor [13]. Many countries have embraced inclusive finance as an essential tool for achieving sustainable development goals (SDGs) [13]. However, it is difficult to take into account both inclusiveness and profitability, resulting in the exclusion of low-income people from financial services [14–18].

Against the backdrop of the rapid development of the mobile internet, the increasing popularity of smartphones, and the rapid digitization of the global economy and finance, governments worldwide are accelerating the development of inclusive finance. Countries are increasingly combining digital technologies to offer finance, and the concept of DFI has emerged. DFI is the fourth stage of the financial revolution after the development of microcredit, microfinance, and inclusive finance [19]. Theoretically, the application of digital technologies in finance has the greatest potential to address financial market failure, namely, digital financial inclusion. Compared with traditional finance, DFI provides financial services through digital technology reducing the threshold to finance for low-income people and improving efficiency. DFI satisfies the requirements of accessibility, affordability, comprehensiveness, and business sustainability which hinder traditional financial services development. DFI provides equal opportunities and rights for low-income and disadvantaged groups to enjoy modern financial services [20, 21]. With rapid technological advances, DFI is proliferating. At least 80 countries have launched state-of-the-art DFI services [22]. Among these technologies, mobile payments have improved the convenience and availability of DFI. In China, more than 70% of adults (about 900 million people) have adopted mobile payments, of which 66.5% are in rural areas [23].

Research on the impact of financial income distribution emerged in the 1990s and gradually became a cutting-edge issue in financial theory. Greenwood and Jovanovic studied the relationship between financial services development and income distribution [10]. Subsequently, the extensive research was carried out on financial services development and income inequality but did not reach consensus. Some researchers believe financial services development widens income inequality [24–26]. Other studies found that financial services development shrinks income inequality [27, 28]. Furthermore, some scholars believe that the impact of financial services development on the urban–rural income gap varies across economic systems, industrial structures, and financial market structures [29, 30]. In most cases, the two exhibit an inverted U-shaped relationship [31–33].

With the growing importance of financial inclusion in the economy, there is focus on the relationship between DFI and rural–urban income disparity in China, and the findings are mixed. On the one hand, many studies argue that DFI plays a significant role in reducing rural–urban income inequality [5, 34–36].On the other hand, Zhao reveals that DFI can effectively narrow the rural–urban income gap if the threshold is lowered [37]. and Qiu et al. suggested that the interaction between DFI and the rural–urban income gap has regional variations [38]. These mixed results indicate that more efforts should be paid on understanding the influences of the forces underlying the process of DFI. Some studies discussed and empirically tested the main mechanism of DFI and the urban–rural income gap. The existing research on the influence mechanism mainly addresses the perspective of innovative entrepreneurship [39–41], employment [42], urbanization [43], and human capital [44]. According to Lewis’s theory, the change in employment caused by the change in industrial structure is the main reason for the inverted U-shaped trend of the income gap [45], the main cause of the urban–rural gap is low productivity, and industrial development is the key driving force for realizing real, sustainable growth of rural residents’ income. However, existing research has not paid enough attention to the mechanism of industrial structure upgrading.

Thus, in the context of the rapid development of DFI and the improvement of urban–rural income inequality in China, this paper examines the impact of DFI on urban–rural income, with the panel data from 52 counties of Zhejiang Province, China, from 2014 to 2020. Further, given that industrial prosperity is the driver for achieving the and sustainable growth of rural residents’ income, this study explores the mechanism for optimizing and upgrading industrial structure, by which DFI reduces urban–rural income inequality. The indicators of industrial structure optimization are empirically tested from three dimensions: industrial structure rationalization, industrial structure advancement, and evolution of internal agricultural structure.

**Fig 1:**
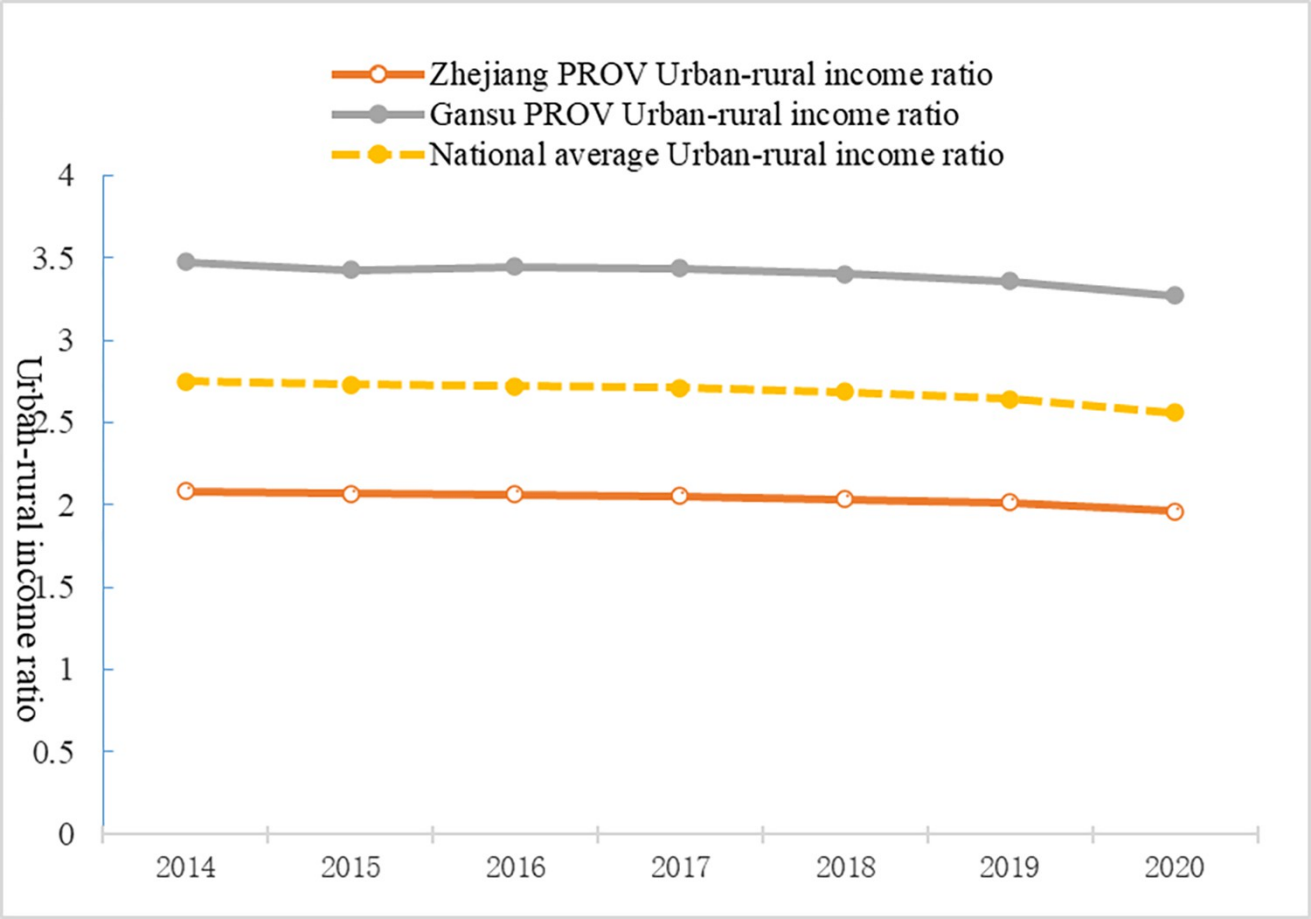
The changes in urban–rural income ratio in China (2014–2020).

As an emerging market economy, China has actively promoted DFI. Differences in economic and social development among China’s regions have led to severe financial exclusion of people in disadvantaged areas. This results in unbalanced and inadequate financial development and clear regional heterogeneity [41, 46]. Unlike existing studies that have used provincial data, this study uses county-level panel data from Zhejiang Province. Zhejiang Province provides an excellent case to clarify the role of DFI in reducing the urban–rural income gap. China has one of the largest urban–rural income gaps [47, 48], and Zhejiang Province has the smallest urban–rural income gap. Zhejiang Province is one of the wealthiest in China, and the income of urban and rural residents in Zhejiang has ranked first among all provinces in China for decades (See Fig 1). The income of urban residents is 1.96 times that of rural residents in Zhejiang in 2020, which is far lower than the national average of 2.56 times (see Fig 1). Furthermore, Zhejiang Province is at the forefront of DFI in China. In 2016, the “G20 High-Level Principles for Digital Financial Inclusion” was released in the provincial capital, Hangzhou. In 2020, the DFI index was 406.88, ranking third in China, second only to Beijing and Shanghai.

This study contributes to the literature in three ways: First, unlike previous research focusing on provincial and municipal levels, this study goes deep into the county level, allowing us to better identify the role of DFI on the urban–rural income gap. Chinese counties are the main battlefield where urban and rural areas integrate and the rural revitalization strategy is implemented. Second, this paper fully considers endogeneity to ensure robust results, which was not done in previous research. Thirdly, this paper explores the mechanism by which DFI helps reduce the urban–rural income gap, which has been rarely discussed in the literature. However, the optimization and upgrading of industrial structures is an important way to bridge the gap between regional income levels. This study provides a new empirical understanding of the mechanism of DFI’s impact on the urban–rural income gap.

The results show that DFI has a significant impact on reducing urban–rural income inequality helps optimize the industrial structure and upgrade the internal structure of agriculture, facilitating income growth for people in rural areas. This study contributes to the current debate on DFI, and provides new empirical evidence from China for in-depth discussions on how developing countries can narrow the urban–rural income gap through DFI. The implications for China and other developing countries is to find feasible ways to cultivate and promote the development of DFI. In addition, the findings of this study from the economically developed provinces in eastern China, which will inform the formulation of DFI policies in less developed regions in central and western China.

The rest of this paper is organized as follows: Section 2 provides theoretical analysis and proposes hypotheses. Section 3 introduces the empirical strategy and data; Section 4 discusses the results and heterogeneity analysis. Section 5 concludes the paper with limitations, implications, and scope for future study.

## Theoretical analysis and hypothesis development

**Fig 2:**
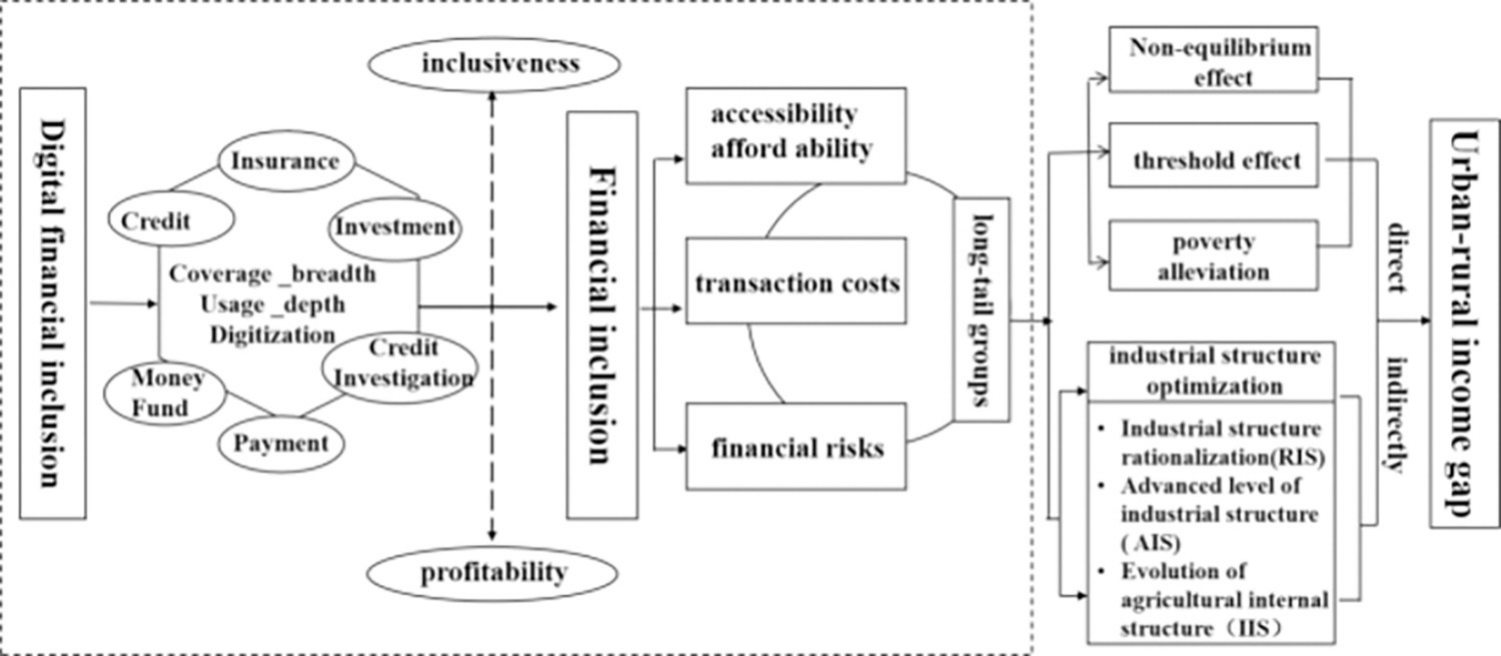
Direct and mediating effects of DFI on urban–rural income disparity.

DFI results from the integration of technology and inclusive finance. As the second-largest economy worldwide, China has broad experience in DFI systems and the proportion of Chinese rural residents accessing financial services is relatively high. According to the "China County Digital Inclusive Finance Index Report 2022", more than half of the surveyed residents have obtained loans, with credit loans accounting for 73.4%. Among them, the proportion of internet loans is the highest at 57.5%. Financial institutions represented by internet banks provide DFI services, which effectively supplement rural finance. DFI provides financial services for low-income long-tail groups. It balances inclusiveness and profitability not offered by traditional financial institutions and promotes sustainable development. DFI narrows urban–rural income disparity through four mechanisms, lowering the threshold for financial services, alleviating poverty, unbalanced financial development, and promoting industrial optimization and upgrading. The mechanisms are shown in Fig 2.

### Direct effects

DFI directly affects the income gap through three mechanisms: (1) the threshold effect of financial development; (2) the effect poverty reduction and income gap; (3) the effect unbalanced financial development and the income gap.

The threshold effect refers to how financial institutions set the price, purchase threshold, collateral, and other thresholds after they consider income and risk. Only customers crossing the threshold can obtain the services. Compared with urban residents, rural residents tend to have lower income, limited wealth accumulation, and a lack of adequate collateral, and they are more sensitive to price or risk. It is challenging to get over the threshold and receive the required financial services. The disequilibrium effect is the premise that financial institutions will prioritize industries or regions with high returns. Compared with cities, economic development in rural areas is lagging, and fewer industries offer high returns. Financial institutions will steer financial resources more to cities, leading to severe financial exclusion in rural areas [49]. The effect of financial development poverty reduction can help poor people escape the "poverty trap" and support low-income groups to catch up by promoting economic growth or providing direct financial support.

In the context of traditional finance, the threshold disequilibrium effects refer to the threshold caused by the unequal allocation of financial resources under the conditions of financial repression, which impacts the urban–rural income gap. The threshold effect and the unbalanced effect of financial services development will generally expand the urban–rural income gap. Providing more financial services to the poor can reduce the income gap and contribute to poverty alleviation. The impact of financial services development on the urban–rural income gap is the synthesis of the three effects.

Compared with traditional finance, DFI provides financial services for low-income long-tail customers through new technologies and transformative business models. DFI provides accessibility, convenience, lower transaction costs, improved risk control and other aspects. It has the following five advantages [19, 50, 51]. First, price advantage, that is, lower costs of obtaining financial services. The supply and demand sides search for product and price information on the platform and complete the transaction. Online transactions reduce the user’s dependence on the physical network, and financial institutions do not need to pay high maintenance fees for the physical network. This reduces the search and transaction cost of the demand side of financial services, as well as the operational costs of the service provider [52]. The second advantage of providing more abundant financial services is that digitalization increases, and applications of the technology are becoming increasingly diversified. The demanders of DFI services need financial investment, payment, insurance, and other types of services, which forces a comprehensive upgrade of financial products and services from the demand side. The third is efficiency, whereby institutions can provide financial services faster. With digital technology, financial institutions can quickly match products and services to the related needs of customers. In addition, financial services can be rapidly connected to many applications, providing information fast to complete the financial products transaction. Fourth is the channel advantage, which is the improved availability of financial services. Thanks to the application of digital technology, DFI reduces geographical restrictions to some extent, expands service boundaries and scope of traditional finance, and enables small and micro enterprises and vulnerable people who were excluded by traditional finance to obtain affordable financial services, which promotes financial inclusion. Fifth, in the risk control advantage, the application of digital technology improves financial institutions’ risk identification and assessment, and provides customers with appropriate financial products to reduce risk. Establishing an effective early warning mechanism can also enable financial institutions and customers to successfully avoid risk in financial service transactions.

Through the above advantages, DFI has brought a new opportunity to solve the shortcomings caused by the " 80/20 rule" of traditional finance, significantly alleviating the contradiction between costs and benefits of inclusive finance. Furthermore, it solves the problem of "mission drift" caused by the difficulty of both inclusive finance and profitability of traditional finance. The "long tail groups", such as rural areas, small and micro enterprises and farmers excluded by traditional finance, can obtain financial services more conveniently and at lower cost, lower the threshold of financial services, alleviate the disequilibrium effect resulting in poverty reduction. Hence, rural areas and relatively poor people can benefit more from the development of digital finance and realize inclusive growth, closing the rural–urban income gap. Therefore, we propose the following hypothesis:

H1: DFI can narrow the urban–rural income gap.

### Mediating effects

The upgrading of industrial structure is an essential factor in the development of a modern economy [53]. Industrial optimization, characterized by the orderly transfer of primary, secondary and tertiary industries, industrial sector and structure upgrades, and the internal evolution of industries, increases economic growth and sustainable development [54]. At the heart of industrial structure optimization is the continuous flow, reallocation and optimization of labor, capital and other factors [55]. Financial services boost the effective allocation of resources and the upgrading of economic structure [56]. Operating efficiency, supply scale and improved structure will narrow the gap between regions regarding science and technology and promote the optimization and upgrading of industrial structure [3, 57].

Innovation in the financial system represented by DFI has reduced information asymmetry and external financing costs of enterprises within and across sectors. The use of algorithms in the rapid upgrading of computer technology, means digital finance can quickly and accurately match both ends of the industrial chain, provide timely funding for industrial development and match digital financial service support with customer needs [58, 59], promoting industrial optimization and upgrading [60]. The convenience, diversity and low cost of DFI services will stimulate consumption. The broadening of residents’ consumption demands and rising consumption levels will drive the optimization and upgrading of industrial structures, encourage agriculture, and promote the emergence of new industries and new business models. Many of these will be derived from traditional agriculture, such as food processing, rural leisure tourism and other new industries. The optimization of the industrial structure promotes the transformation of products from low value to high-value products. It addresses the urban–rural income gap by changing the distribution of economic output between capital, labor and other factors. With optimization, the remuneration of rural labor flowing to local industrial and service sectors or part-time jobs will increase correspondingly, and the urban–rural income gap resulting from differences in remuneration between sectors will be reduced; As agricultural industry is upgraded, the urban–rural income gap will be reduced by utilizing agricultural surplus labor, improving agricultural operation efficiency, and increasing farmers’ incomes.

To sum up, DFI can reduce financial exclusion in rural areas by optimizing the allocation of financial resources and creating conditions for upgrading industrial structure in rural areas, thereby promoting employment, entrepreneurship and innovation This means that rural residents can share the benefits of industrial structure optimization such as the integration of rural industries, and multiple income channels to make their income grow faster than urban residents, thus narrowing the income urban–rural income gap [61–63]. Therefore, the following hypothesis is proposed:

H2: DFI indirectly promotes the narrowing of the urban–rural income gap by optimizing industrial structure.

## Methodology and data

### Empirical model

The empirical model is presented in Eq (1):

$${URID}_{it}=\beta_{0}+\beta_{1}{DFI}_{it}+\varphi X_{it}+\mu_{i}+\gamma_{t}+\varepsilon_{it}$$ (1)

Where *i* is the region, *t* is the time, *URID*_*it*_ represents the urban–rural income gap. *DFI*_*it*_ is the key variable, indicating the development level of DFI. *X*_*it*_ is the vector of other control variables, including per capita GDP, local fiscal budget expenditure, urbanization rate, and residents’ education level. *u*_*i*_ represents country-fixed effect, *γ*_*i*_ denotes unobservable time effect, and *ε*_*it*_ is the random error term.

The above theoretical analysis shows that optimizing industrial structure may be an important mechanism for DFI to reduce the urban–rural income gap. To verify this hypothesis, we set Eq (2):

$${URID}_{it}=\beta_{0}+\beta_{1}{DFI}_{it}+\beta_{2}{industry}_{it}+\beta_{3}{DFI}_{it}\times {industry}_{it}+\varphi X_{it}+\mu_{i}+\gamma_{t}+\varepsilon_{it}$$ (2)

Where industry is the level of industrial structure optimization, the meaning of other variables is the same as that of Eq (1).

### Measurement of variables

#### Measurements of URID variables

Previous studies have used different indicators to measure URID, including the Gini coefficient, gap ratio, and Theil index [64–66]. Although the Gini coefficient can estimate URID, it is not neatly decomposable by subgroup. Compared with the gap ratio, the Theil index is a more comprehensive indicator because it captures the income ratio of urban and rural households and reflects changes in the urbanization rate. Given its significant benefits, the Theil index has been widely applied in research [65–67]. This study employed the Theil index to measure URID at the county level.

#### Independent variable: Digital financial inclusion

The study used the financial inclusion index jointly prepared by the Digital Finance Research Center of Peking University and Ant Financial Services Group. The process is shown in Guo et al. [58]. The index has been widely used in recent years because of its underlying data, including segmentation, and continuous publishing. The index includes one total index and three primary indexes. This paper selects the financial inclusion index of each county to measure the overall level of local DFI development and also analyzes the impact of the coverage, depth and digitalization of digital finance on the urban–rural income gap.

#### Industrial structure optimization

The optimization and upgrading of the industrial structure reflect the rationalization of the industrial structure, the enhancement of economic serviceability, and the internal collaborative evolution of the industry. This indicates the improvement of industrial efficiency and the rational allocation of the industrial structure. Following Du et al. [68] and Gan et al. [69], we develop industrial structure optimization to measure industrial structure optimization in three dimensions:

Industrial structure rationalization (RIS). RIS shows the aggregation of different industries, the degree of coordination between industries and the efficiency of resource use. It measures the synergy of industrial input factors and output structure. Therefore, this study draws on the RIS index proposed by Qiao [70]. Rationalization is a solid foundation for upgrading, and upgrading away can easily lead to a "virtual high" of industrial structure. For rationalization, the adjusted Thiel index takes into account the position of each industry in the economy and effectively avoids the calculation of absolute value. We use the following equation to measure RIS:

$$TL=\sum_{k=1}^{n}\left(\frac{Y_{K}}{Y})ln(\frac{Y_{K}}{L_{K}}/\frac{Y}{L}\right)$$ (3)

In Eq (3), *TL* is the adjusted Thile index, *n* represents the number of departments, *K* stands for industry, *Y*_*K*_ represents the added value of each industry, *L*_*K*_ represents the number of employees in each industry, *Y* and *L* represents total output value and total population, respectively. For convenience, we take the reciprocal logarithm as the RIS index. The larger the index value, the more rationalized the industrial structure.

Industrial structure advancement (AIS). AIS reflects the upgrading of the industrial structure develops from the low end to the middle and high end, which is reflected in the gradual development of the primary industry to secondary and tertiary industries, and the development of products from low value to high value. Because in the context of all-around penetration of information technology and economic service, “service-oriented industrial structure” is an important feature of AIS, we follow most of the literature and measure AIS by the ratio of tertiary industry and secondary industrial regional GDP [71].

Evolution of internal agricultural structure (IIS). IIS is formed by the complex and nonlinear interaction within the industry [72]. In China, the important position of agriculture determines that the optimization of county industrial structure mostly depends on the integration and extension of agriculture-based industries and the upgrading of the agricultural internal structure. For example, the transformation from single agriculture dominated by agricultural product supply to multi-function agriculture, such as leisure and ecological agriculture has strengthened the tendency for division of labor and agricultural service within agriculture. Thus, following Li and Huang, the internal evolution of agricultural industrial structure at county level is measured by the internal evolution of the agricultural industry as a whole [73]. This study take the ratio of output value of professional and support activities for agriculture, forestry, animal husbandry and fishery to the gross output value of agriculture, forestry, animal husbandry and fishery as a proxy indicator of IIS.

#### Other variables

We considered data availability and drew on existing income inequality literature [31, 40, 43, 57, 74, 75] for the control variables. Seven control variables were selected: GDP, urbanization rate, public expenditure, education, loan depth, and population density. Table 1 presents detailed definitions of the selected variables.

**Table 1:** Descriptive statistics.

Types	Variables	Observations	Mean	Standard deviation
**Explained variable**	Urban–rural income disparity (URID)	363	0.049	0.016
**Explanatory variables**	The DFI development level	363	109.639	16.251
	Coverage of digital financial inclusion (Breadth)	363	102.468	10.578
	Usage of DFI (Depth)	363	137.553	30.753
	Degree of digitization	363	82.711	37.903
**Mediator variables**	Industrial structure rationalization (RIS)	363	2.465	1.273
	Industrial structure advancement (AIS)	363	1.121	0.552
	Evolution of internal agricultural structure (IIS)	363	0.024	0.026
**Control variables**	The ratio of urban population (Urbanization)	363	0.390	0.155
	Traditional financial development (Deposit)	363	1.293	0.300
	Highway mileage (Roads)	363	34.586	21.918
	Education level (Education)	363	2.613	1.670
	Local fiscal budget expenditure (Fiscal)	363	0.198	0.120
	Logarithm of GDP per capita (PGDP)	363	11.096	0.400
**instrumental variable**	geographical features, Distance to Hangzhou (IV)	51	135.253	78.487

(1) The real GDP per capita (PGDP) was used to control the impact of economic development on urban–rural income disparity. Using the per capita GDP of each county to measure the level of economic development, we deflated the monetary variables to the 2011 base year using the consumer price index; (2) Urbanization is driven by the structural shift from agriculture to modern industrial and service sectors [76], which may narrow urban–rural income disparity. The argument that China’s urban–rural dual economic structure is the main reason for the urban–rural income gap is widely supported, so it is included as the control variable. (3) Traditional financial development is measured by the ratio of the credit balance of financial institutions to GDP. (4) Human capital (Education). Education directly affects workers’ productivity and wage level and is an important factor affecting the urban–rural income gap. Education is the main way to improve the level of human capital. Given the availability of data, the number of ordinal college students per 10000 people was used to measure education level. (5) Local government expenditures (Fiscal). Local fiscal expenditure has the function of distribution and redistribution, which impacts the urban–rural income gap. It is measured by the proportion of general public budget expenditure to regional. (6) Transportation infrastructure (Roads). Transportation infrastructure can affect the speed and convenience of flows between urban and rural areas, and affect the urban–rural income gap. We used highway mileage in the county as a proxy for transportation infrastructure. (7) Geographical features (IV). Following Guo et al., the geographic distance from the sample county to Hangzhou was selected as instrumental variable [58]. The linear distance from the center point of the sample county (city) to the center point of Hangzhou City was calculated using the geographic information system (GIS). Table 1 summarizes the descriptive statistics of the variables used in the model.

### Study area and data

**Fig 3:**
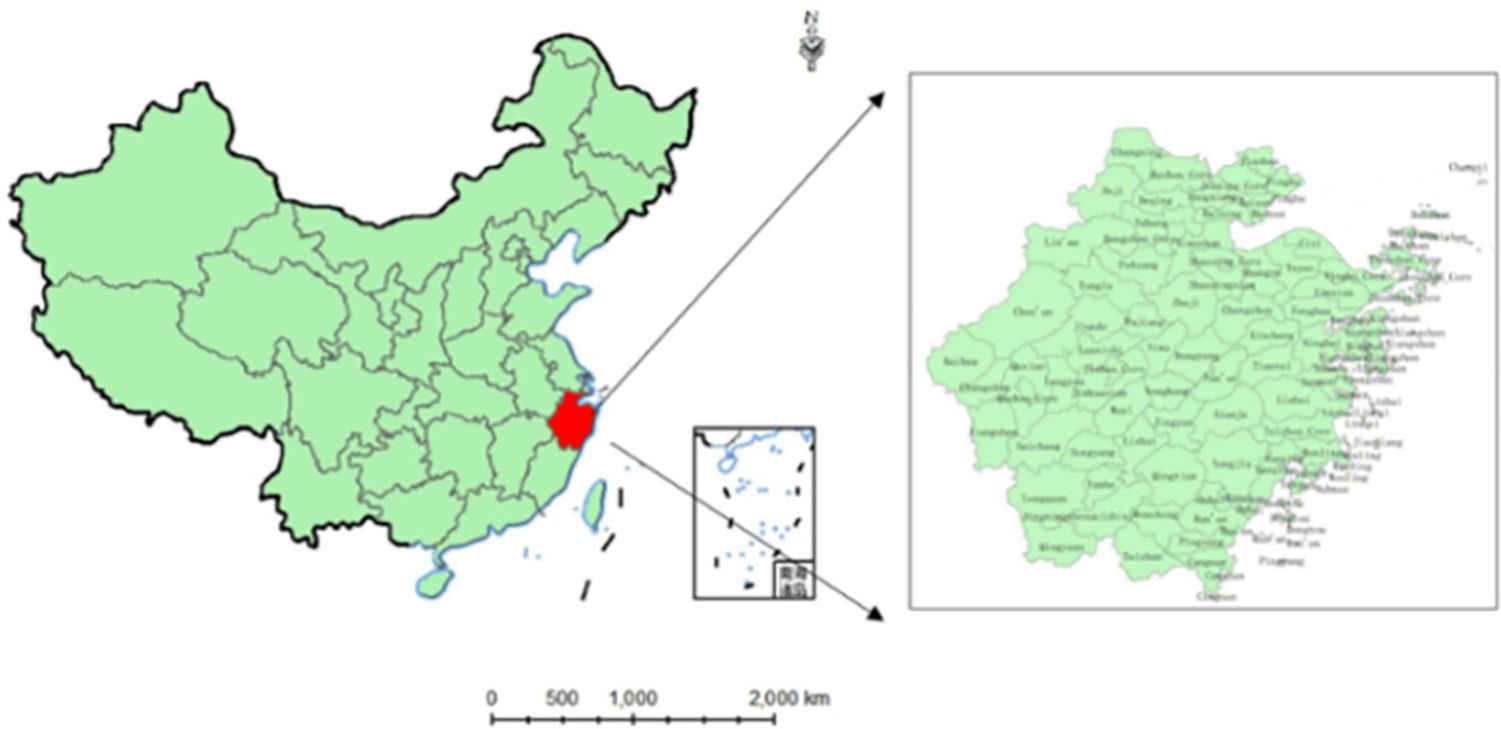
Geographical location and administrative division map of Zhejiang Province, China.

Zhejiang Province is located on the east coast of China, at the intersection of the Belt and Road Initiative and the Yangtze River Economic Belt (see Fig 3). There are two main reasons for choosing the Zhejiang Province for this study. First, Zhejiang Province has made remarkable achievements in exploring and solving the problem of unbalanced and inadequate development, which to some extent epitomizes the development gap between urban and rural areas in eastern China. The income of urban and rural residents in Zhejiang has ranked first among all provinces in China for 20 years and 36 years, respectively. The urban–rural income ratio in 2020 was 1.96, which is much lower than the national level. In 2021, the CPC Central Committee and The State Council entrusted Zhejiang with a major strategic mission to develop high-quality construction of shared prosperity as a demonstration area, providing a provincial example for the national promotion of common prosperity. Narrowing the income gap between urban and rural areas was its main aim. Secondly, Zhejiang originated DFI, which is at the leading level in China. Therefore, it is the ideal place to test the correlation between DFI and urban and rural income.

Considering that the economic development urbanization of municipal districts is generally high, many municipal districts have been managed as urban areas. Statistical data of the population and regional GDP of some municipal districts are missing, and so 52 county-level administrative regions2 were taken as research objects.

Data used in this paper were collected from three major sources: the provincial and county-level Zhejiang Statistical Yearbooks, the Peking University DFI Index of China (PKU—DFIIC), and the National Geomatics Center of China. The Hangzhou distance data was measured using GIS based on the 1:4 million electronic maps provided by the National Geomatics Center of China. Data for DFI were collected from the PKU—DFIIC This county-level data is available from 2014. Therefore, our research period begins in 2014. Other data are from the Zhejiang Provincial Statistical Yearbook and the Municipal Statistical Yearbook. Our final dataset included information on 52 counties from Zhejiang Province in China for 2014–2020, with 363 samples in total.

## Results

### Baseline results

The panel data was analyzed using fixed and random effects models. We also employed a Hausman test. The benchmark regression results show that the p-value was 0.0115. The fixed effect model should be used instead of the original hypothesis. Therefore, this paper used a fixed effect model to estimate Eqs (1) and (2), and shows the estimation results of the random effect model and mixed panel least squares (OLS) as a comparison. Table 2 reports the results.

**Table 2:** Digital financial inclusion and urban–rural income disparity: Baseline results.

Variables	OLS		Fixed effects model		Random effects model	
	(1)	(2)	(3)	(4)	(5)	(6)
**DFI**	-0.001***	-0.000	-0.000*	0.000***	-0.000*	0.000***
	(-0.000)	(-0.000)	(-0.000)	(-0.000)	(-0.000)	(-0.000)
**Urbanization**		-0.003		0.010		0.008
		(-0.013)		(-0.010)		(-0.010)
**Deposit**		-0.010*		0.003		0.000
		(-0.005)		(-0.003)		(-0.003)
**Agricultural output ratio**		-0.005		-0.002		0.000
		(-0.006)		(-0.005)		(-0.003)
**Roads**		0.000		0.000*		0.000
		(-0.000)		(-0.000)		(-0.000)
**Education**		-0.004**		0.004**		-0.001
		(-0.001)		(-0.002)		(-0.001)
**Fiscal**		-0.035*		0.003		0.005
		(-0.021)		(-0.017)		(-0.017)
**PGDP**		0.002		0.009***		0.007***
		(-0.005)		(-0.003)		(-0.002)
**Constant**	0.098***	0.055	0.058***	-0.0510*	0.0641***	-0.016
	(-0.017)	(-0.047)	(-0.008)	(-0.029)	(-0.0124)	(-0.026)
**Year fixed effects**	Yes	Yes	Yes	Yes	Yes	Yes
**County fixed effects**	Yes	Yes	Yes	Yes	Yes	Yes
**Observations**	363	363	363	363	363	363
**R** ^**2**^	0.666	0.743	0.507	0.584		

Agricultural output ratio is agricultural output as a proportion of total output.

The t-test statistics are in parentheses [

*** p<0.01

** p<0.05

* p<0.1].

Column (3) in Table 2 shows the results of the independent regression of DFI to the URID. The coefficient of the DFI index is significantly negative at the 10% level, indicating that the DFI helps narrow the urban–rural income gap, and the result persists after adding control variables. This result supports hypothesis H1. From the fixed effects regression results (Column 4 of Table 2), the urban–rural income gap (Theil index) decreases by 0.003 for each unit of DFI increase. The results of the control variables, the level of infrastructure construction, regional economic development and education are significantly positively correlated with reducing the urban–rural income gap.

#### Results of the digital financial inclusion sub-index model

China’s DFI index has three aspects: coverage, depth of use, and degree of digitalization. OLS, fixed effect and random effect were used to add other control variables o examine the relationship between the DFI sub-indexes and the urban–rural income gap and not to add control variables for regression. The results are shown in Table 3. The results of the three methods are relatively robust, and the fixed effect model with other control variables is mainly used in later analysis. The regression results in column (4) of Table 3, show a significant negative impact on the urban–rural income gap after controlling for the variables, coverage, depth of use and degree of digitalization of finance. In terms of coefficient size, their impact on the urban–rural income gap is equivalent. Improving the coverage and depth of digital finance reflects improvements in integrating digital technology and inclusive financial services. Financial services have broken geographical and market barriers, lowered the threshold of financial services, increased financial coverage, and alleviated rural financial exclusion, thereby improving rural inhabitants’ income levels and narrowing the income gap with urban residents.

**Table 3:** Result of the sub-index model.

Variables	OLS		Fixed effects model		Random effects model	
	(1)	(2)	(3)	(4)	(5)	(6)
**Breadth**	-0.000**	-0.000	-0.000	-0.000**	-0.000	0.000***
	(-0.000)	(-0.000)	(-0.000)	(-0.000)	(-0.000)	(-0.000)
**Depth**	-0.000**	0.000	-0.000	-0.000**	-0.000	-0.000
	(-0.000)	(-0.000)	(-0.000)	(-0.000)	(-0.000)	(-0.000)
**Digitization**	-0.000	-0.000	0.000**	0.000***	-0.000**	-0.000**
	(-0.000)	(-0.000)	(0.000)	(0.000)	(0.000)	(0.000)
**Urbanization**		-0.002		0.010*		0.008
		(-0.007)		(-0.005)		(-0.005)
**Deposit**		0.011***		0.004		0.000
		(-0.003)		(-0.003)		(-0.003)
**Road**		0.000***		0.000***		0.000**
		(-0.000)		(-0.000)		(-0.000)
**Education**		-0.004***		0.005***		-0.001
		(-0.001)		(-0.002)		(-0.001)
**Fiscal**		-0.032***		0.002		0.005
		(-0.011)		(-0.014)		(-0.011)
**PGDP**		0.001		0.009***		0.007***
		(-0.003)		(-0.003)		(-0.002)
**Constant**	0.102***	0.051*	0.059***	-0.052*	0.067***	-0.016
	(-0.013)	(-0.030)	(-0.009)	(-0.028)	(-0.010)	(-0.025)
**Year fixed effects**	Yes	Yes	Yes	Yes	Yes	Yes
**County fixed effects**	Yes	Yes	Yes	Yes	Yes	Yes
**Observations**	363	363	363	363	363	363
**R** ^**2**^	0.67	0.745	0.511	0.588		

Agricultural output ratio is agricultural output as a proportion of total output.

The t-test statistics are in parentheses [

*** p<0.01

** p<0.05

* p<0.1].

### Results of mediation regression analysis

The theoretical analysis shows that an important aim for DFI is to narrow the urban–rural income gap by promoting the optimization of industrial structure. To verify this, interaction terms were used. We used several methods to test the robustness of the model. The level of RIS measures the optimization of the industrial structure and the internal evolution of rural industries. The regression results are shown in Tables 4–6.

**Table 4:** DFI, RIS and urban–rural income disparity.

Variables	Hybrid OLS		Fixed effects model		Random effects model	
**DFI**	-0.000	0.001**	0.000	-0.000	-0.000	-0.000
	(-0.000)	(-0.000)	(-0.000)	(-0.000)	(-0.000)	(-0.000)
**RIS**	0.003	0.014***	0.011***	0.008***	0.010***	0.008***
	(-0.004)	(-0.004)	(-0.002)	(-0.002)	(-0.002)	(-0.002)
**DFI×RIS**	-0.000**	-0.000***	-0.000***	-0.000***	-0.000***	-0.000***
	(0.000)	(0.000)	(0.000)	(0.000)	(0.000)	(0.000)
**Control variables**	No	Yes	No	Yes	No	Yes
**Year fixed effects**	Yes	Yes	Yes	Yes	Yes	Yes
**County fixed effects**	Yes	Yes	Yes	Yes	Yes	Yes
**Observations**	312	312	312	312	312	312
**R** ^**2**^	0.38	0.523	0.467	0.523		

The t-test statistics are in parentheses [

*** p<0.01

** p<0.05

* p<0.1].

**Table 5:** DFI, AIS and urban–rural income disparity.

Variables	Hybrid OLS		Fixed effects model		Random effects model	
**DFI**	-0.001***	0.000	-0.000**	-0.000***	-0.000***	-0.000**
	0.000	-0.000	-0.000	-0.000	-0.000	-0.000
**AIS**	-0.027***	-0.019	-0.002	0.003	-0.002	-0.000
	-0.007	-0.017	-0.005	-0.005	-0.005	-0.005
**DFI×AIS**	0.000***	0.000	0.000	0.000	0.000	0.000
	-0.000	-0.000	0.000	0.000	0.000	0.000
**Control variables**	No	Yes	No	Yes	No	Yes
**Year fixed effects**	Yes	Yes	Yes	Yes	Yes	Yes
**County fixed effects**	Yes	Yes	Yes	Yes	Yes	Yes
**Observations**	363	363	363	363	363	363
**R** ^**2**^	0.213	0.515	0.508	0.582		

The t-test statistics are in parentheses [

*** p<0.01

** p<0.05

* p<0.1].

**Table 6:** DFI, IIS and urban–rural income disparity.

Variables	OLS		Fixed effects model		Random effects model	
**DFI**	-0.001	0.001	-0.000	-0.000***	-0.000*	-0.000**
	0	-0.000	-0.000	-0.000	-0.000	-0.000
**IIS**	0.076	0.489**	0.215	0.176	0.049	0.02
	-0.219	-0.217	-0.164	-0.155	-0.137	-0.132
**DFI×IIS**	-0.002	-0.005**	-0.002**	-0.002*	-0.002*	-0.001
	-0.002	-0.002	-0.001	-0.001	-0.001	-0.001
**Control variables**	No	Yes	No	Yes	No	Yes
**Year fixed effects**	Yes	Yes	Yes	Yes	Yes	Yes
**County fixed effects**	Yes	Yes	Yes	Yes	Yes	Yes
**Observations**	312	312	312	312	312	312
**R** ^**2**^	0.226	0.497	0.402	0.484		

The t-test statistics are in parentheses [

*** p<0.01

** p<0.05

* p<0.1].

#### Digital financial inclusion, industrial structure rationalization and urban–rural income disparity

Table 4 shows that the coefficient of the interaction between the rationalization of industrial structure and the financial inclusion index is significantly negative at the 1% level, and the coefficient of the rationalization of industrial structure is positive. This shows that DFI is conducive to rationalizing the industrial structure and reducing the income gap between urban and rural areas. Research hypothesis 2 is therefore supported. The rationalization of industrial structure is essentially the optimal allocation of regional endowments to regional industries, with labor force as the most important factor. When the selected industries of a region are consistent with the comparative advantages conferred by regional endowments, the economy is most competitive, and the income distribution of residents will be balanced [77]. Suppose a single industry is given priority for development. In that case, the industrial and employment structures may deviate, leading to surplus population in the agricultural sector who cannot be quickly absorbed by urban industries or new rural forms of business This means the rural surplus labor force cannot be effectively allocated, which will expand the urban–rural income gap.

#### Digital financial inclusion, industrial structure improvements and urban–rural income disparity

This paper examines the role of industrial structure improvement reflected by the ratio (logarithm) of the service industry to the second industry in the impact of DFI on the urban–rural income gap. Table 5 shows that the interaction coefficient between the financial inclusion index and the advanced industrial structure variable is insignificant. The coefficient of the advanced variable of industrial structure is not significant. This means that DFI has not narrowed the income gap through industrial structure upgrades. DFI failed to promote the significant service-oriented tendency of the county industrial structure. This may be because county economies in Zhejiang are driven by primary and secondary industries, and the service industry is relatively lagging. The transformation of the tertiary industry has failed to impact the urban–rural income gap in the short term. This is similar to the results of Zhang & Guo [78] on transforming the employment structure and urban–rural income gap.

#### Digital financial inclusion, the internal structure of the agricultural industry and urban–rural income disparity

As shown in Table 6, the coefficient of the interaction term between the DFI index and the internal structure of the agricultural industry is significantly negative at the 1% significance level, indicating that the DFI has promoted changes to the internal structure of the agricultural industry, reducing the urban–rural income gap. In China’s rural economy, the important position of agriculture drives the optimization of county-level industries, especially in rural areas, which rely on agriculture-based industrial integration and the upgrading of agricultural internal structures. As an early and active adopter of digital technology, Zhejiang has seen its county-level agricultural industry evolve in recent years, helped by digital technologies such as e-commerce and the process of agricultural service-driven industrial upgrading and rural industrial integration. DFI provides financial support for agriculture-based industrial integration, producing increased income and narrowing the urban–rural income gap.

To sum up, the contribution of the county industrial structure to reducing the urban–rural income gap is mainly through the RIS and the evolution of the internal structure of the agricultural industry, and the role of industrial upgrading is much smaller.

### Heterogeneity analysis: Different regions

The results show that the development of DFI has narrowed the urban–rural income gap, but it is not known whether regions at different economic development levels can enjoy the dividends of digital finance equally. Is DFI more helpful in narrowing the urban–rural income gap in underdeveloped counties? Is there any difference in the mechanism of industrial structure optimization? To answer these questions, the sample group was further divided into 26 counties in mountainous areas and the sample group of non-mountainous counties (26 counties). The estimation Formula (1) is respectively estimated in the two samples to test regional heterogeneity of the impact of DFI on the urban–rural income gap. The results are shown in Table 7. The regression results of different models are relatively stable.

**Table 7:** Heterogeneity analysis of different regions.

Variables	Mountainous counties			Non-mountainous counties		
	OLS (1)	FE (2)	RE (3)	OLS (4)	FE (5)	RE (6)
**DFI**	-0.000	-0.000**	-0.000**	0.000***	-0.000*	-0.000**
	-0.000	-0.000	-0.000	-0.000	-0.000	-0.000
**Urbanization**	0.058***	0.043**	0.043**	-0.008	0.003	0.001
	-0.019	-0.020	-0.021	-0.009	-0.009	-0.009
**Deposit**	0.001	0.017***	0.012**	-0.005*	-0.005*	-0.006***
	-0.006	-0.005	-0.006	-0.003	-0.003	-0.002
**Agricultural output ratio**	-0.015*	-0.016	-0.009	-0.003	0.003	0.001
	-0.008	-0.012	-0.007	-0.004	-0.002	-0.003
**Roads**	0.000	0.000	0.000	-0.000	-0.000	-0.000
	-0.000	-0.000	-0.000	-0.000	-0.000	-0.000
**Education**	-0.003	0.007***	0.002	0.004***	-0.001	-0.004***
	-0.002	-0.002	-0.002	-0.001	-0.002	-0.001
**Fiscal**	-0.051	-0.034*	-0.031*	0.024	0.047**	0.048**
	-0.037	-0.018	-0.018	-0.028	-0.018	-0.020
**LogPGDP**	0.009	0.004	0.005	0.002	0.010***	0.009**
	-0.006	-0.004	-0.004	-0.004	-0.004	-0.004
**Constant**	-0.022	0.012	0.026	0.066	-0.062	-0.033
	-0.070	-0.045	-0.049	-0.051	-0.047	-0.044
**Year fixed effects**	Yes	Yes	Yes	Yes	Yes	Yes
**County fixed effects**	Yes	Yes	Yes	Yes	Yes	Yes
**Observations**	160	160	160	203	203	203
**R** ^**2**^	0.671	0.698		0.874	0.697	

The t-test statistics are in parentheses [

*** p<0.01

** p<0.05

* p<0.1].

#### Direct effects

Table 7 shows the inclusion index’s coefficient is significantly negative in both mountainous and non-mountainous counties, and the development of digital finance has promoted the urban–rural income gap within their respective regions. However, the regression coefficient (-0.000) of the DFI index in the mountainous counties (the second column of Table 6) is smaller than that in non-mountainous counties (-0.000) (the fifth column of Table 6), which confirms that relatively poor counties benefit more from the development of digital finance, reflecting the impact of digital finance in income growth. Our results support H2. However, the estimated coefficients of traditional finance in both mountainous and non-mountainous counties are significant but in the opposite direction, showing the unbalanced effect of traditional finance. Traditional finance has significantly reduced the urban–rural income gap in non-mountainous counties, but has expanded the urban–rural income gap in the 26 mountainous counties. This confirms that the relatively backward regions are excluded due to the unbalanced effect of traditional finance analyzed by the previous theory. Based on the above analysis, the effect of traditional finance on the urban–rural income gap is objective, and the development of digital finance has effectively made up for this shortcoming.

#### The intermediary role of industrial structure

The previous analysis shows that digital finance reduces the urban–rural income gap by optimizing and upgrading industrial structure. If DFI improves the income of people in backward areas through industrial development, then increasing DFI should promote industrial development in relatively backward areas, making up for the shortcomings of traditional finance and reducing the gap between urban and rural areas. To verify this, we used Formula (2) in two samples of 26 counties in mountainous areas and 26 in non-mountainous areas. The results are shown in Tables 8–10.

**Table 8:** DFI, RIS and urban–rural income disparity in different regions.

Variables	Mountainous counties			Non-mountainous counties		
	OLS (1)	FE (2)	RD (3)	OLS (4)	FE (5)	RD (6)
**DFI**	0.001*	0.000**	0.000*	-0.000	0.000	0.000
	(-0.001)	(0.000)	(0.000)	(0.000)	(0.000)	(0.000)
**RIS**	-0.000	0.004	0.005	0.005	0.007***	0.006***
	(-0.012)	(-0.006)	(-0.006)	(-0.004)	(-0.002)	(-0.002)
**DFI×RIS**	0.000	-0.000	-0.000	0.000*	0.000***	0.000***
	(0.000)	(0.000)	(0.000)	(0.000)	(0.000)	(0.000)
**Control variables**	Yes	Yes	Yes	Yes	Yes	Yes
**Year fixed effects**	Yes	Yes	Yes	Yes	Yes	Yes
**County fixed effects**	Yes	Yes	Yes	Yes	Yes	Yes
**Observations**	138	138	138	174	174	174
**R** ^**2**^	0.472	0.788		0.542	0.409	

The t-test statistics are in parentheses [

*** p<0.01

** p<0.05

* p<0.1].

**Table 9:** DFI, AIS and urban–rural income disparity in different regions.

Variables	Mountainous counties			Non-mountainous counties		
	OLS	FE	RD	OLS	FE	RD
**DFI**	0.001	-0.000*	-0.000	-0.001***	-0.000	-0.000
	(-0.001)	(-0.000)	(-0.000)	(-0.000)	(-0.000)	(-0.000)
**AIS**	-0.024	0.005	0.001	-0.001	0.004	0.002
	(-0.017)	(-0.012)	(-0.013)	(-0.010)	(-0.004)	(-0.004)
**DFI×AIS**	0.000*	-0.000	0.000	0.000	-0.000	-0.000
	(-0.000)	(-0.000)	(-0.000)	(-0.000)	(0.000)	(0.000)
**Control variables**	Yes	Yes	Yes	Yes	Yes	Yes
**Year fixed effects**	Yes	Yes	Yes	Yes	Yes	Yes
**County fixed effects**	Yes	Yes	Yes	Yes	Yes	Yes
**Observations**	160	160	160	203	203	203
**R** ^**2**^	0.447	0.68		0.533	0.696	

The t-test statistics are in parentheses [

*** p<0.01

** p<0.05

* p<0.1].

**Table 10:** DFI, IIS and urban–rural income disparity in different regions.

Variables	Mountainous counties			Non-mountainous counties		
	OLS	FE	RD	OLS	FE	RD
**DFI**	0.001**	-0.000**	-0.000*	-0.000	-0.000	-0.000
	(-0.000)	(-0.000)	(-0.000)	(-0.000)	(-0.000)	(-0.000)
**IIS**	0.759	0.999**	0.872**	-0.089	-0.066	-0.123
	(-0.788)	(-0.459)	(-0.415)	(-0.186)	(-0.135)	(-0.123)
**DFI×IIS**	-0.007	-0.007**	-0.008**	-0.001	-0.000	0.000
	(-0.006)	(-0.003)	(-0.003)	(-0.001)	(-0.001)	(-0.001)
**Control variables**	Yes	Yes	Yes	Yes	Yes	Yes
**Year fixed effects**	Yes	Yes	Yes	Yes	Yes	Yes
**County fixed effects**	Yes	Yes	Yes	Yes	Yes	Yes
**Observations**	138	138	138	174	174	174
**R** ^**2**^	0.472	0.798		0.567	0.354	

The t-test statistics are in parentheses [

*** p<0.01

** p<0.05

* p<0.1].

As shown in Table 8, the coefficient of the financial inclusion index and the rationalization of industrial structure is not significant in mountainous areas, but is significantly negative in the sample of other counties at the 1% level. This shows that DFI reduces the income gap by promoting the RIS effectively in non-mountainous counties, but not significantly in mountainous counties. It can also be seen that the industrial structure of other economically developed counties is more reasonable than mountainous areas. The rationalization of industrial structure is an important reason for the difference in economic development between the two regions.

Table 9 shows the coefficient of the DFI index and industrial structure upgrading is not significant in mountain counties, indicating that digital finance has not played a role in promoting industrial upgrading and has not reduced the urban–rural income gap.

Table 10 shows the coefficient of financial inclusion and the evolution of the internal structure of the agricultural industry is significantly negative at the 1% level in mountainous counties, but not significant in other counties. This shows that financial inclusion has promoted the internal evolution of the agricultural industry in mountainous counties, thus narrowing the income gap. The low impact in non-mountainous counties may be because there is more agriculture in mountainous areas than in non-mountainous areas. DFI significantly promotes the development of agriculture, forestry, animal husbandry and fishery services.

In general, the effect of DFI on the urban–rural income gap in relatively backward counties is more significant. Different dimensions of industrial structure optimization show heterogeneity in the two sample groups. In relatively poor counties, digital finance reduces the urban–rural income gap through the development of the structure of the agricultural industry; In relatively developed counties, digital finance reduces the urban–rural income gap through rationalizing the industrial structure.

### Endogeneity and robustness checks

Individual fixed effect, time fixed effect and related variables at county level were controlled in the baseline regression to reduce the impact of missing variables on the results. We found a statistically significant association between DFI, industrial structure optimization, and reducing urban–rural income disparity within a region. However, widening the urban and rural income gap may increase the urban -rural digital divide, resulting in reverse causality. To address this, we employed generalized method of moments (GMM) estimations with IV to identify the effect of DFI.

The IV is the geographical distance from the sample counties to Hangzhou measured by GIS. The reasons for using this measurement are as follows: First, the development of digital finance by Alipay originated in Hangzhou, which is correlated with the development degree of digital finance in the sample counties. As the compilation of the PKU—DFIIC is mainly from Alipay data, Hangzhou, as the location of the Alibaba Group, can represent the development level of digital. The closer it is geographically to Hangzhou, the better the development level. Second, the geographical distance from the sample counties to Hangzhou is an exogenous variable, which meets the requirements of instrumental variables [58]. Further, the development of digital finance changes over time, but the geographical distance from each county to Hangzhou does not vary.

The geographical distance from each county to Hangzhou was interacted with the DFI index value of Zhejiang Province, and a time variable was constructed. The GMM method was used for estimation, and the results are shown in Table 11. From the results, we can see the coefficient of DFI is still significantly negative in counties in mountainous areas, indicating that the development of DFI has indeed played a role in narrowing the urban–rural income gap.

**Table 11:** IV Results.

Variables	IV-FE	Mountainous counties	Non-mountainous counties
**DFI**	-0.000***	-0.001***	-0.000***
	(-0.000)	(-0.000)	(-0.000)
**Urbanization**	0.010*	0.046***	0.003
	(-0.006)	(-0.013)	(-0.005)
**Deposit**	0.003	0.017***	-0.005*
	(-0.003)	(-0.005)	(-0.003)
**Agricultural output ratio**	-0.002	-0.015*	0.003
	(-0.005)	(-0.008)	(-0.005)
**Roads**	0.000***	0.000**	-0.000
	(-0.000)	(-0.000)	(-0.000)
**Education**	0.004***	0.007***	-0.002
	(-0.002)	(-0.002)	(-0.002)
**Fiscal**	0.002	-0.031	0.047**
	(-0.014)	(-0.020)	(-0.022)
**PGDP**	0.009***	0.005	0.011***
	(-0.003)	(-0.006)	(-0.003)
**Control variables**	-0.050*	0.008	-0.060*
	(-0.029)	(-0.057)	(-0.036)
**Year fixed effects**	Yes	Yes	Yes
**County fixed effects**	Yes	Yes	Yes
**Observations**	363	160	203

The t-test statistics are in parentheses [

*** p<0.01

** p<0.05

* p<0.1].

#### Robustness checks

In the previous section, we employed various approaches to examine the effects of DFI on urban–rural income disparity and identify the mechanisms through which such effects operate. First, mixed OLS, fixed effect and random effect models were used for group sample estimation. the conclusions obtained by the three methods are consistent with the main estimation results, indicating that the results in this study are robust. The results with and without major control variables were also estimated in the main regression. The core explanatory variables are the same, indicating that the estimation results are robust. Due to length limitations, the robustness test results of other methods will not be shown in the paper.

## Conclusions

We used the county-level panel data of Zhejiang Province in China from 2014 to 2020, with a double fixed effect and GMM to study the impact of DFI on the urban–rural income gap, and further examined the impact of different dimensions of industrial structure optimization on the relationship between the two. The conclusions are as follows: Firstly, the development of DFI has promoted the narrowing of the urban–rural income gap in Zhejiang, China. After the robustness test, these findings are still valid. Secondly, further research on the mechanism of action shows that DFI has reduced the urban–rural income gap by promoting the optimization of industrial structure. Specifically, the contribution of the county industrial structure to the reduction of the urban–rural income gap is mainly the RIS and the evolution of the internal structure of the agricultural industry. The role of upgrading the industrial structure is much smaller. Thirdly, the heterogeneity analysis shows that the convergence of digital finance on the urban–rural income gap in relatively poor counties is more significant. In relatively poor counties, digital finance promotes the narrowing of urban–rural income gap mainly through the internal evolution of agricultural industrial structure; In relatively developed counties, DFI mainly converges the urban–rural income gap through rationalizing industrial structure.

## Discussion and policy recommendations

The inequality in urban and rural development is a fundamental issue affecting China’s national and regional development. Since ancient times, there has been a saying in China that "the country is governed by prefectures and counties, and the world is safe". It is more meaningful to narrow the urban–rural income gap within the county to reduce overall regional inequality and achieve regional coordinated development because the geographical environment of urban and rural areas within the county are similar. This paper draws attention to rural–urban income disparity at the county level. As China’s urbanization has entered a stable development stage, and the focus in rural and urban areas has shifted to control of the gap. However, China currently sees the county seat as an important carrier for new urbanization, supporting township integration. Narrowing the gap between urban and rural areas in the county will become a key breakthrough for solving urban and rural problems and promoting regional coordinated development. In this sense, our results based on county analysis have important practical significance for revitalizing rural areas and achieving regional sustainable development. The results of this study provide clear policy implications for narrowing the urban–rural income gap.

(1) The development of DFI plays a positive role in reducing the urban–rural income gap and sustainable development goals. Further, improving the digital infrastructure, encouraging financial institutions, and science and technology enterprises to strengthen digital financial innovation, explore the convergence of digital technology and inclusive finance, promote the establishment of an innovative DFI system with lower costs, risk sharing, revenue sharing, and stronger price discovery function, and encourage financial institutions to develop financial products for farmers, take poverty alleviation as the leading direction to promote rural revitalization, use local design, digital products, and services to upgrade digital platforms to empower rural industries, and consolidate the sustainable growth of rural residents’ income by increasing productivity. In particular, we should focus on guiding financial services to focus on rural areas, farmers and other "long-tail groups", narrow the income gap, and achieve common prosperity. At the same time, government departments and financial and non-financial institutions should disseminate financial knowledge and education, especially strengthening educational support for poor areas such as financial and internet education for vulnerable groups, and making comprehensive efforts from both demand and supply sides to eliminate financial exclusion.

(2) Building a diversified rural industrial system is the key to narrowing the urban–rural income gap. Optimizing and upgrading the rural industrial structure, expanding the agricultural boundaries, and diversifying rural industries are effective measures to improve the income of rural residents and mitigate the risk of income fluctuations. First, agricultural services should be expanded, and industrialization and modernization should be promoted through agricultural structural adjustment. Further, improvements in agricultural productivity and economic returns should be made by expanding the scale of the entire agricultural supply chain and improving the division of labor. This is the path of agricultural modernization that China, with more people and less land, should take. The optimization of rural industrial structure is important, to fully explore and expand the functions of agriculture, accelerate the integration of agriculture and the modern service and information technology industry, promoting the integration of primary, secondary and tertiary industries.

(3) The problem of unbalanced development between regions will be alleviated by implementing financial and industrial policies. Counties should be encouraged to formulate industrial development policies based on local conditions, regional comparative advantages, and resource endowments. The optimization and upgrading of industrial structure in relatively poor counties should focus on promoting the evolution and upgrading and extending the internal system of the agricultural industry, and the integration of secondary and tertiary industries to narrow the urban–rural income gap. In relatively developed counties, more attention should be paid to the role of rationalization of industrial structure in the convergence of urban–rural income gap channels.

In this study, restricted by the availability of data and the compatibility of data from different organizations, the DFI index of Peking University has selected the Ant Financial Services Group as the sole data source, it fails to cover the impact of traditional financial institutions with greater contributions. so it cannot depict the development landscape of China’s digital financial inclusion in a comprehensive manner. This may underestimate the development level of China’s DFI. In the later research, we hope to compile a financial inclusion index that includes the banking system and use more reliable instrumental variables to study DFI in China more comprehensively.
